# Molecular Profiling and FTIR Characterization of Wheat Germ Oil, Supported by the Screening of Its Anti-Inflammatory and Cytotoxic Properties

**DOI:** 10.3390/biom15040464

**Published:** 2025-03-21

**Authors:** Paweł Paśko, Agnieszka Galanty, Emilia Ramos-Zambrano, Alma Leticia Martinez Ayala, Mikołaj Gralak, Joanna Gdula-Argasińska, Danail Pavlov, Joseph Deutsch, Shela Gorinstein

**Affiliations:** 1 Department of Food Chemistry and Nutrition, Faculty of Pharmacy, Jagiellonian University Medical College, 30-688 Kraków, Poland; p.pasko@uj.edu.pl; 2 Department of Pharmacognosy, Faculty of Pharmacy, Jagiellonian University Medical College, 30-688 Kraków, Poland; agnieszka.galanty@uj.edu.pl; 3 Centro de Desarrollo de Productos Bióticos, Instituto Politécnico Nacional, Yautepec 62731, Mexico; ailimerz@hotmail.com (E.R.-Z.); almarayala@hotmail.com (A.L.M.A.); 4 Department of Physiological Sciences, Warsaw University of Life Sciences—SGGW, 02-787 Warsaw, Poland; mikolaj_gralak@sggw.edu.pl; 5 Department of Radioligands, Faculty of Pharmacy, Jagiellonian University Medical College, 30-688 Kraków, Poland; joanna.gdula-argasinska@uj.edu.pl; 6 Department of Biochemistry, Molecular Medicine and Nutrigenomics with Laboratory of Nutrigenomics, Functional Foods and Nutraceuticals, Faculty of Pharmacy, Medical University “Prof. Dr. Paraskev Stoyanov”, 9002 Varna, Bulgaria; danailpavlov@gmail.com; 7 Institute for Drug Research, School of Pharmacy, Faculty of Medicine, The Hebrew University, Jerusalem 9112002, Israel

**Keywords:** wheat germ oil, squalene, fatty acids, FTIR, anti-inflammatory effect, cytotoxic potential, human protein binding

## Abstract

Wheat germ oil (WGO), derived from the nutrient-dense germ of wheat kernels, is a functional bioactive product, known for its rich composition of essential fatty acids, sterols, tocopherols, and polyphenols. This study aimed to comprehensively profile the molecular and therapeutic properties of WGO, focusing on its antioxidant, cytotoxic, and anti-inflammatory activity. Using advanced analytical techniques such as gas chromatography-mass spectrometry (GC-MS), Fourier Transform Infrared (FTIR) spectroscopy, and fluorescence analysis, WGO was shown to contain high levels of linoleic acid (45.3%), squalene (2.52 g/100 g), and polyphenols. WGO displayed selective cytotoxicity, inhibiting cancer cells’ viability in melanoma, prostate, and colorectal cancer cell lines, but not normal cells, highlighting its chemoprevention potential. Furthermore, WGO significantly reduced LPS-induced nitric oxide and IL-6 production in macrophages, with effects plateauing at higher doses. The 3D fluorescence spectra showed a significant decrease in fluorescence intensity when human serum albumin interacted with the WGO polyphenol fraction, indicating a strong binding affinity and stable complex formation. These findings emphasize the nutritional and therapeutic potential of WGO as a natural bioactive agent, warranting further mechanistic investigation and broader applications in health and disease management.

## 1. Introduction

Wheat germ oil (WGO), extracted from the germ of wheat (*Triticum aestivum* L.) kernels, is a highly valued natural product, with an exceptional nutritional profile and a wide range of health-promoting properties. The germ, a small yet biologically rich portion of the wheat grain, constitutes approximately 2% of the grain’s total weight and is separated from the endosperm during milling, to avoid unfavorable baking characteristics [1]. This by-product of the milling industry, often used for animal feed or oil production, contains a wealth of bioactive compounds, including tocopherols, sterols, carotenoids, and phenolic substances, which contribute to its numerous health benefits [1]. Wheat germ oil is primarily composed of unsaturated fatty acids such as linoleic acid (omega-6), oleic acid (omega-9), and a smaller proportion of α-linolenic acid (omega-3), alongside saturated fatty acids, such as palmitic acid [2]. These fatty acids are of high importance in maintaining cardiovascular and overall metabolic health. Additionally, WGO contains a variety of lipid classes, including glycolipids, phospholipids, and nonpolar lipids, enhancing its nutritional and functional versatility [3]. One of the most remarkable features of WGO is its exceptionally high tocopherol content, particularly of α-tocopherol, which accounts for up to 60% of its tocopherol profile, and contributes significantly to its antioxidant capacity [4]. Tocopherols protect cellular structures against oxidative damage and support overall cellular health. Moreover, the presence of phytosterols, such as β-sitosterol and campesterol, adds cholesterol-lowering and anti-inflammatory properties to the oil’s bioactive profile [4,5]. Phenolic compounds present in WGO further enhance its ability to reduce oxidative stress, thus mitigating cellular damage and supporting longevity [6]. Beyond its nutritional and therapeutic benefits, WGO also contains volatile compounds, such as esters, alcohols, and aldehydes, which contribute to its distinct aroma and may hold additional therapeutic potential [7]. The major volatile constituents are hexanal, 2-methyl-2-butene, 2,4-heptadienal, and limonene. Due to this impressive composition, WGO has gained attention as a functional food ingredient, capable of improving the polyunsaturated fatty acid (PUFA) content, and optimizing the omega-3-to-omega-6 ratio in food products [4]. Additionally, this oil is suggested to be a source of squalene; however, until now, no quantitative analysis on its amount has been performed [8]. Despite such a recognizable composition, WGO is still the source of unexplored antioxidants and anti-inflammatory compounds [9]. It should be highlighted that studies on wheat germ oil are relatively rare, as most research focuses on wheat bran oil and its processed forms, such as fermented wheat germ oil or defatted wheat germ oil or their extracts. Additionally, many studies emphasize the nutritional and bioactive properties of its components rather than the raw oil itself as a final product [10,11].

Wheat germ oil has demonstrated significant potential as a functional ingredient in food, cosmetics, and pharmaceuticals, owing to its antioxidant and cholesterol-lowering properties [12]. However, additional exploration into its biological activities, particularly its cytotoxic and anti-inflammatory effects, is essential to unlock its full potential.

This study aims to provide a comprehensive molecular profile of wheat germ oil by characterizing its bioactive components, such as squalene, fatty acids, and polyphenols, and evaluating its therapeutic potential. The research utilizes advanced analytical techniques, including Fourier Transform Infrared (FTIR) spectroscopy and three-dimensional fluorescence analysis, to investigate the chemical composition and molecular signatures of WGO. Additionally, this study explores its antioxidant and anti-inflammatory properties, alongside with cytotoxic effects on prostate, gastrointestinal, and melanoma cancer cell lines. Finally, the safety and selectivity of WGO are evaluated, using non-cancerous cell lines, to assess its potential as a chemopreventive agent.

## 2. Materials and Methods

### 2.1. Wheat Germ Oil (WGO)

Wheat germ, separated from the endosperm during the milling of wheat harvested in Germany in 2023, was powdered and sieved (100-mesh sieve). A total of 100 g of the powdered sieved material was extracted with n-hexane (500 mL) at 60 °C for 4 h, using the Soxhlet apparatus in three repetitions. The resulting extracts were evaporated, and the samples were stored at −20 °C for subsequent experiments. The separation of wheat germ oil (WGO) into lipophilic and hydrophilic fractions was performed using Tween 80 (Polysorbate 80, Sigma-Aldrich, St. Louis, MO, USA) from 20 to 25% and 0.9% NaCl solution in a 1:1:10 ratio (oil–Tween 80–NaCl). The components were thoroughly mixed and vortexed to ensure proper emulsification. The mixture was then subjected to centrifugation (5000–6000 rpm for 15–20 min) to facilitate phase separation. The lipophilic fraction (oil-rich phase), containing nonpolar components such as triglycerides, sterols, and fat-soluble vitamins, formed the upper layer due to its lower density. The hydrophilic fraction (water phase), containing polar compounds like phospholipids and water-soluble antioxidants, remained at the bottom. Since Tween 80 acts as an emulsifier, it may stabilize the oil–water interface, requiring either centrifugation or increased NaCl concentration to enhance phase separation by disrupting the emulsion [13,14].

### 2.2. Determination of Fatty Acid Profile in WGO

WGO was extracted by the Folch method, using a chloroform–methanol mixture (2:1 *v*/*v*), with the addition of butylated hydroxytoluene (0.005%) as an antioxidant. Fatty acid methyl esters (FAMEs) were prepared by methylation using 20% BF_3_ in methanol at 60 °C. FAME analysis was conducted using gas chromatography on an Agilent 6890 N gas chromatograph (Agilent Technologies, Santa Clara, CA, USA), with a DB-23 capillary column ((50%-Cyanopropyl)-methylpolysiloxane, 60 m, ID 0.25 mm, film thickness 0.25 μm) and a flame ionization detector (FID). The detailed analytical procedure has been described in previous work [15]. Fatty acid identification was performed with relation to the retention times of a standard mix of fatty acid methyl esters (Supelco 47801 Sigma-Aldrich, St. Louis, MO, USA). The results, averaged from three replicates, were expressed as total fatty acid content percentages.

### 2.3. Analysis of Squalene Concentration in WGO

Gas chromatography coupled with mass spectrometry (GC-MS) was used to identify and quantify squalene in the WGO samples. For this, 20 μL of WGO was diluted directly in 1 mL of hexane and taken for analysis. Helium as the carrier gas was used at a constant flow rate of 1 mL/min. The oven temperature program started at 60 °C (held for 1 min), followed by a gradual increase of 10 °C per minute until reaching 255 °C, where it was held for 15 min. The sample injection volume was set at 2 μL, with the MS source temperature maintained at 230 °C and the quadrupole temperature at 150 °C. A TG-5MS column from Thermo Scientific (Whatman, MA, USA) was used, featuring a length of 60 m, an internal diameter of 0.25 mm, and a film thickness of 0.25 μm. Squalene quantification was carried out using a calibration curve prepared specifically for squalene.

We have omitted the saponification step, which, as reported by Popa et al. [16], may lead to the degradation of bioactive compounds such as squalene and tocopherols when analyzed by GC. By implementing these methodological considerations, we minimize compound loss and improve quantification reliability. Furthermore, previous studies have demonstrated that GC-MS provides satisfactory results in terms of repeatability, sensitivity, linearity, and recovery, as reported by Cowan et al. [17] and Santivañez et al. [18].

### 2.4. Antioxidant Activity and Total Phenolic Content in WGO

The total antioxidant capacities of WGO, along with its hydrophilic and lipophilic fractions, were evaluated using the CUPRAC (Cupric Reducing Antioxidant Capacity) assay, as detailed in previous studies [19]. The CUPRAC method employs the copper (II)-neocuproine [Cu(II)-Nc] complex as a chromogenic oxidizing agent. The extracts or standard solutions, along with water, were mixed with Cu(II), Nc, and an NH4Ac buffer solution. The absorbance of the resulting solution was measured (450 nm) in relation to a reagent blank. The results were presented as the micromole Trolox equivalent (TE) per 1 mL of oil. The total phenolic (TPC) content in WGO and its hydrophilic and lipophilic fractions were quantified using the Folin–Ciocalteu method, as outlined by Benito-Román et al. [20]. Following oil extraction and centrifugation, the supernatants were processed using a methanol–water mixture (80/20, *v*/*v*). The resulting methanolic extracts were then combined with Folin–Ciocalteu reagent, sodium carbonate solution, and distilled water. After being incubated in the dark for 240 min, the absorbance was measured at 765 nm using a Jasco V-530 spectrophotometer (Tokyo, Japan). The TPC content was expressed as µg of gallic acid equivalents (GAE) per 1 mL of oil sample. All experiments were repeated in three independent replicates.

### 2.5. Fourier Transform Infrared Spectroscopy

FTIR analysis of WGO samples in Attenuated Total Reflectance (ATR) mode was performed with an IRAffinity-1S spectrometer (Shimadzu, Tokyo, Japan), utilizing the ATR accessory. Spectra were recorded in the absorbance mode across the mid-infrared region (4000–500 cm^−1^), with 90 scans per sample at a resolution of 4 cm^−1^. Data acquisition and processing were carried out using FTIR Spectroscopy Software LabSolutions IR (Shimadzu, Tokyo, Japan) (https://www.shimadzu.com/an/products/molecular-spectroscopy/ftir/ftir-spectroscopy-software/labsolutions-ir/index.html, accessed on 16 March 2025). Tannic acid, gallic acid, quercetin, and squalene were employed as reference materials for comparison.

### 2.6. Cytotoxic Activity of WGO

The cytotoxic potential of wheat germ oil (WGO) was evaluated on various human cancer and non-cancerous cell lines, purchased from Merck (Darmstadt, Germany). These included the following: (1) prostate cell lines such as DU-145 (androgen-insensitive, grade IV prostate carcinoma derived from a metastatic brain site), PC-3 (androgen-insensitive prostate carcinoma derived from a metastatic bone site), LNCaP (androgen-sensitive prostate adenocarcinoma derived from a lymph node metastatic site), and normal prostate epithelial cells, PNT2; (2) gastrointestinal cell lines such as colorectal adenocarcinomas Caco-2 and HT-29, and hepatocellular carcinoma HepG2; and (3) skin cell lines, including melanoma cell lines HTB140 (derived from a lymph node metastasis), A375 (malignant melanoma), and HaCaT keratinocytes. All cell lines were cultured under standard conditions (37 °C, 5% CO_2_, relative humidity) using appropriate media. The WGO samples were accurately weighed and then diluted in culture media to achieve final working concentrations ranging from 0 to 100 μg/mL starting from freshly prepared DMSO stock solutions (10 mg/mL). Cell viability was assessed using the LDH assay, as previously described [15]. Briefly, 1.5 × 10^4^ cells/well were seeded into 96-well plates and incubated overnight. Afterward, the medium was replaced with the fresh one, and WGO (2.5–100 μg/mL) was added. After 24 h, the absorbance was measured according to the manufacturers’ protocol. All tests were conducted in triplicate, and results were expressed as percentages of cell viability (mean ± SD) alongside IC_50_ values (the concentration inhibiting 50% of cell viability). DMSO control was also included. Doxorubicin was used as the reference drug for comparison.

### 2.7. Anti-Inflammatory Activity of WGO

First, the impact of the tested oil on RAW 264.7 cell viability was tested using the LDH assay, as described above, to assess non-toxic doses of WGO. The anti-inflammatory activity of WGO was assessed using RAW 264.7 murine macrophages, seeded in 96-well plates at a density 1.5 × 10^5^ cells/well and pre-treated with WGO at concentrations of 5 and 20 μg/mL for one hour, prepared by weighing the oil and dispersing it in the culture medium. Subsequently, lipopolysaccharide (LPS, 10 ng/mL) was added to initiate the inflammatory process, as described by Paśko et al. [15]. Dexamethasone (0.5 μg/mL) was employed as a reference anti-inflammatory agent. DMSO control was also included. After a 24 h incubation period, the cells’ supernatants were collected for further analysis.

### 2.8. Nitric Oxide Release

The nitric oxide (NO) levels in RAW 264.7 cells’ supernatants were measured using the Griess reagent kit (Promega Corporation, Madison, WI, USA) according to the manufacturer’s instructions. The analysis of WGO’s influence on NO release was conducted in triplicate, with absorbance measurement conducted on the BioTek Synergy microplate reader (BioTek Instruments Inc., Winooski, VT, USA). Results were expressed as a percentage relative to the LPS control.

### 2.9. TNF-Alpha and IL-6 Analysis

Cytokine levels, specifically, those of TNF-alpha and IL-6, were quantified using human ELISA kits (Bioassay Technology Laboratory, Shanghai, China) following the manufacturer’s instructions. RAW 264.7 cells’ supernatants were analyzed in triplicate using a BioTek Synergy microplate reader, with results presented as a percentage of the LPS control.

### 2.10. Fluorometric Measurements

Fluorometric techniques were utilized to assess the binding properties of wheat germ oil (WGO) to human serum albumin (HSA). Both two-dimensional (2D-FL) and three-dimensional (3D-FL) fluorescence spectra were recorded for WGO prepared by accurately weighing WGO and dissolving it in an appropriate solvent to achieve a concentration of 0.17 mg/mL using an FP-6500 spectrofluorometer (Jasco, Tokyo, Japan), equipped with a 1.0 cm quartz cuvette and a thermostatic bath. For 2D-FL measurements, emission spectra were collected over a range of 310 to 500 nm, with excitation set at 295 nm. The 3D-FL spectra were obtained by sequentially scanning emission wavelengths from 200 to 550 nm in 1.0 nm increments, while varying the excitation wavelength from 200 to 500 nm in 10 nm increments. Gallic acid and epicatechin were used as reference standards [21]. A reaction mixture containing 2.0 × 10^−5^ mol/L HSA in a 0.05 mol/L Tris–HCl buffer with 0.1 mol/L NaCl (pH 7.4) was prepared. The binding properties of WGO to HSA were evaluated by calculating the percentage change in fluorescence intensity at key peaks (a and b). These values were determined by comparing the fluorescence intensity of the HSA solution before and after interaction with WGO. This approach provided insights into the interaction between WGO and HSA, reflecting the potential of WGO as a bioactive agent.

### 2.11. Statistical Analysis

All experiments were performed in triplicate, and the results are presented as mean values accompanied by their standard deviations (SDs). The data obtained for WGO samples were subjected to statistical analysis using one-way analysis of variance (ANOVA) followed by Tukey’s post hoc test to assess differences between groups. Statistical calculations were performed using STATISTICA v. 13.3 (TIBCO Software Inc., Palo Alto, CA, USA). A *p*-value of ≤ 0.05 was considered indicative of statistically significant differences among the groups.

## 3. Results and Discussion

### 3.1. Fatty Acid Profile and Squalene Content

The fatty acid composition of wheat germ oil (WGO) (Table 1) revealed a predominance of linoleic acid (C18:2 n-6), constituting 45.3% of the total fatty acids, followed by oleic acid (C18:1 n-9), a monounsaturated fatty acid contributing 26.8% to the total profile. Palmitic acid (C16:0), a saturated fatty acid, was present at 19.4%, while stearic acid (C18:0) contributed a minor portion at 4.6%. Notably, α-linolenic acid (C18:3 n-3), an omega-3 fatty acid, was found at 3.9%, further enhancing the oil’s nutritional profile. Our results about fatty acid profile in WGO are in agreement with Ghafoor et al. [6], who noted linoleic acid, which comprises 42–59%, followed by palmitic (11–17%) and oleic acid (14–25%), as predominates. The saturated stearic acid is usually less than 2%, and linolenic acid comprises 4–12% of the fatty acid contents of WGO.

Squalene is represented in significantly smaller quantities in plant oils, when compared to its animal abundance, and can be found in olive, Amaranthus, palm, rice-bran, wheat germ oil [8]. However, in the available literature, we could not find any data on the content of squalene in WGO. The only report, by Niu et al. [4], indicating its presence together with other unsaponifiable substances, such as campesterol, β-sitosterol, and fucosterol, was based on a qualitative analysis. This prompted us to perform a quantitative analysis of squalene in WGO, resulting in the determination of its content as 2.52 g/100 g of oil. The obtained amount of squalene in WGO was significantly lower than that in amaranth oil (7.6 g/100 g), but the level was comparable to that in buckwheat oil (2.1 g/100 g) [15]. Our results, for the first time, indicate that WGO may be another rich plant source of squalene.

### 3.2. Antioxidant Activity and Total Phenolic Compunds

The antioxidant activity of WGO itself, but also its lipophilic and hydrophilic fractions, was measured using the CUPRAC assay and was presented as mM Trolox equivalents per milliliter (Table 1). The total antioxidant activity of the oil was 0.38 ± 0.07 mM TE/mL. The antioxidant activity of the hydrophilic fraction was significantly higher, at 0.24 ± 0.04 mM TE/mL, highlighting the contribution of higher levels of polyphenols, which are potent antioxidants, while the lipophilic (fat-soluble) fraction is rich in tocopherols (vitamin E). Studies have shown that the hydrophilic fraction exhibits stronger antioxidant activity due to its higher polyphenol content [9,22]. The lipophilic fraction, as mentioned above, contains fat-soluble compounds such as squalene or fatty acids. The lipophilic fraction showed a lower activity of 0.13 ± 0.05 mM TE/mL, suggesting fewer water-soluble antioxidants in the oil.

The total polyphenol content in the oil was 7.40 ± 0.8 µg GAE/mL (Table 1). The hydrophilic fraction exhibited a significantly higher polyphenol concentration of 4.69 ± 0.70 µg GAE/mL, underscoring the role of polyphenols in the antioxidant properties of WGO.

According to Małecka et al. [23], wheat germ oil contains a high concentration of tocopherols, which are key contributors to its antioxidant properties. Specific sterols, such as Δ-5-avenasterol and citrostadienol, enhance the oil’s antioxidant activity due to the ethylidene bond in their side chains. The presence of squalene in wheat germ oil, indicated in our study, adds to its antioxidant capacity, especially during heating processes, while polyphenols may also contribute depending on their thermal stability. The oil’s antioxidant activity is effective in protecting against oxidative damage, making it valuable for health and food preservation.

### 3.3. Fourier Transform Infrared Spectroscopy

**Figure 1:**
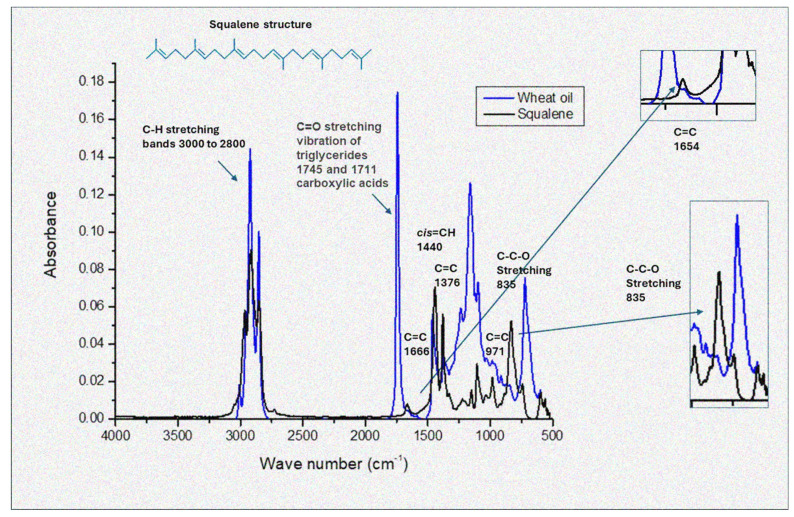
FTIR spectra in the range 4000–500 cm^−1^ of wheat germ oil combined with squalene as reference material. For each point, 3 samples were analyzed and are shown in the figure. *y*-axis = absorbance, *x*-axis = wavelength (cm^−1^).

FTIR is recognized as a rapid, nondestructive, and cost-effective analytical method that provides insight into the chemical composition and physical state of total lipids during oil analysis [24,25]. In this study, FTIR absorption spectra were utilized to identify similarities in the content of lipids, sterols, phenols, and flavonoids in WGO, compared to squalene, quercetin, tannic acid, and gallic acid as reference standards. The results are presented in Figure 1.

In Figure 1, some characteristic features can be seen, such as the bands at 3008 cm^−1^ (symmetric stretching of the cis double bond, =CH); shoulder at 2954 cm^−1^ (asymmetric C–H stretching of aliphatic CH_3_ groups due to the remaining alkyl of triglycerides present in large amounts of vegetable oils); bands at 2923 cm^−1^ (asymmetric C–H vibration of aliphatic group CH_2_); bands at 2854 cm^−1^ (symmetric C-H stretching of aliphatic groups CH_2_ and CH_3_); bands at 1745 cm^−1^ (carbonyl group stretching of triglycerides, C=O); shoulder at 1711 cm^−1^, (carbonyl group stretch of free fatty acids, C=O), whose band presents a broad signal, indicating a high abundance of fatty acids; bands at 1666 cm^−1^, (cis double bond stretch of disubstituted olefins, RCH=RCH); bands at 1440 cm^−1^ (C–H bending of aliphatic groups CH_2_ and CH_3_); bands at 1376 cm^−1^ (C=C); bands at 1237, 1161, 1118, and 1096 cm^−1^ (stretching of C–O groups); bands at 971 cm^−1^ (out-of-plane bending of trans bonds of disubstituted olefins –HC=CH–); and bands at 835 cm^−1^ (C–C–O stretching).

The bands characteristic for squalene are observed at 1440 cm^−1^ (C–H bending of aliphatic CH_2_ and CH_3_ groups); 1666 cm^−1^ (stretching of cis double bonds of disubstituted olefins, RCH=CHR); 2850 cm^−1^ (symmetrical stretching of C–H of aliphatic CH_2_ and CH_3_ groups); 2925 cm^−1^ (asymmetric vibration of C–H of aliphatic CH_2_ group of methylenes), present in fats; and 2954 cm^−1^ (asymmetric stretching of C–H of CH_3_ groups of methyls). These bands highlight the presence of double bonds within the squalene structure, which also characterizes some fatty acids found in wheat oil.

The spectra of the wheat oil showed substantial similarity to sesame oil, as reported by Deng et al. [26], but displayed significant differences when compared to pseudocereal oils [15].

The FTIR spectral analysis confirmed the absence of polyphenols in the wheat germ oil and highlighted its distinct lipid profile, dominated by triglycerides and squalene. This finding aligns with the therapeutic potential of WGO, as documented in the literature. Future studies should explore the relationship between these spectral properties and the bioactivity of WGO.

The FTIR spectra of WGO also revealed characteristic peaks that reflect its rich lipid profile. The bands at 1744 cm^−1^ (carbonyl stretching vibration of triglycerides) align closely with observations from other studies on lipid-rich oils. For instance, the study by Arslan et al. [27] noted similar spectral features in the triglyceride ester linkage and carbonyl group, with a prominent peak at 1746 cm^−1^, further supporting the presence of fatty acids and triglycerides in WGO.

The strong peak at 720–730 cm^−1^ observed in WGO, associated with the –(CH_2_)n out-of-plane rocking mode, also reflects its saturated lipid components. This pattern is consistent with findings in sesame oil, as noted by Deng et al. [26], emphasizing the structural similarities among plant-derived oils. However, subtle differences in the band intensities between WGO and pseudocereal oils [15] suggest variability in the lipid chain length and saturation levels.

Squalene, a key bioactive component in WGO, exhibited overlapping spectral characteristics with the oil. The peaks at 2925 cm^−1^ and 2850 cm^−1^, corresponding to the C–H stretching of methyl and methylene groups, are indicative of its unsaturated hydrocarbon structure. These bands were similarly prominent in the WGO spectra, confirming the presence of squalene within the lipid matrix. The structural resemblance between squalene and other fatty acids in WGO further highlights its unique role as a source of biomolecules with the potential for health benefits.

Unlike tannic acid, gallic acid, and quercetin standards, WGO displayed a distinct lack of strong peaks associated with aromatic rings and hydroxyl groups, which are characteristic of polyphenols. This is evidenced by the absence of intense bands in the region <1500 cm^−1^, such as those found in quercetin and tannic acid due to C–O and O–H stretching vibrations. As noted by Arslan et al. [27], the presence of peaks at 1238 cm^−1^ and 1033 cm^−1^, associated with –C–O stretching vibrations and aromatic structures, is a hallmark of polyphenolic compounds; we observed only a peak at 1237 but the second one was notably absent in WGO.

The fingerprint region (1500–650 cm^−1^) of WGO demonstrated significant differences compared to those of tannic and gallic acid. These findings contrast with the more complex spectral profiles observed in pseudocereal oils [15], where phenolic and aliphatic components coexist. We confirmed that WGO exhibited antioxidant activity in both the hydrophilic and lipophilic fractions of the oil. However, the lack of typical elements for polyphenols in the FTIR analysis suggests a new direction in the search for other hydrophilic antioxidants besides polyphenols. It can be clarified that FTIR mainly detects dominant functional groups present in high concentrations (e.g., ester bonds in triglycerides, characteristic bands of squalene). In contrast, polyphenols may not be clearly visible due to their unique chemical structures or interactions with lipids.

### 3.4. Cytotoxic Potential of WGO

**Figure 2:**
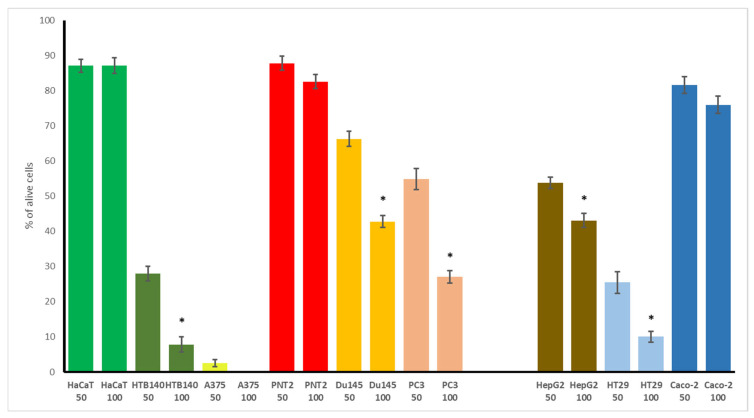
Cytotoxic activity of wheat germ oil (WGO) on non-cancerous and cancer cells in the skin, prostate, and gastrointestinal panel. Cells were incubated with 50 and 100 μg/mL of WGO. Cell viability was expressed as % of control (untreated) cells (n = 3). The significant differences (*p* < 0.05) between used concentration in each cell line are marked with asterisks (*).

In the first step of evaluating WGO’s bioactivity, its cytotoxic potential was screened using a number of cancer and normal cells, representing the skin, prostate, and gastrointestinal tract. This was justified by the most common use of other plant oils, easily applied on the skin, often used as an element of the diet, or for treating some prostate disorders. The results, presented in Figure 2 (for the two highest concentrations tested) and in Table 2, as IC_50_ values, indicated that WGO was not toxic to the non-cancerous cells examined (HaCaT and PNT2), maintaining a high percentage of cell viability (87.2% and 82.6%, respectively). In contrast, the cancer cells demonstrated varying degrees of sensitivity to WGO. In the skin panel, the viability of the A375 melanoma cell line was completely inhibited at the highest concentration tested, with an IC_50_ of 18 μg/mL, and also, a dramatic reduction in the viability of highly metastatic melanoma HTB140 (7.8%) was noted. Interesting results were observed in the prostate panel, with the most profound effect found for the androgen-dependent prostate cancer LNCaP cells (IC_50_ 15.4 μg/mL), while the Du145 and PC3 cells were more resistant. However, the highly metastatic PC3 cells were more vulnerable to WGO than the less aggressive DU145 cells. The gastrointestinal cancer cells such as HT29 (IC_50_ 25.2 μg/mL) and HepG2 (43% viability) were also strongly affected, while the Caco-2 cells were more resistant.

The most important observation from this part of our study is the high selectivity of WGO to non-cancerous cells, with marked effectiveness observed on some of the cancer cells, which is also in contrast to the non-selective effect of doxorubicin.

Although a number of studies have been published on the effect of wheat germ extract on different normal and cancer cells, only scarce data exist on the cytotoxic potential of WGO. These include its impact on human breast cancer MCF7 cells, with IC_50_ 1.03 mg/mL [28], or the decrease in the viability of human cervical cancer HeLa and glioblastoma U97 cells, observed at a concentration of 1 mg/mL, while lung cancer A549 cells were not affected [29]. Thus, our results indicate a significant, few-fold higher cytotoxic effect, as compared to that reported in the studies performed so far. Interestingly, our data are in agreement with those of Gömeç et al. [30], who reported no toxic effect of WGO on murine L929 fibroblasts at a concentration up to 100 ng/mL. Moreover, the authors proved the in vitro wound-healing effect of WGO on the examined fibroblasts, observed as an increase in cell proliferation and enhanced cell migration within the wound area [30]. This suggests that WGO is characterized by appropriate safety towards non-cancerous skin cells, while our study also demonstrated its high effectiveness against different melanoma cancer cells. All these indicate that the potential topical use of WGO should be examined more intensively in further studies. Moreover, the results of our study are the first to demonstrate the effectiveness of WGO on androgen-dependent LNCaP prostate cancer cells, and also its slightly lower effect on highly aggressive prostate cancer PC3 cells, with no toxicity towards normal prostate epithelial cells. This seems to be another prospective direction of future studies on WGO. Plant oils, rich in bioactive compounds such as phytosterols, polyphenols, and unsaturated fatty acids, may play a protective role in prostate cancer by modulating inflammatory pathways, oxidative stress, and hormone metabolism. Certain components, like omega-3 fatty acids and plant-derived sterols, have been shown to inhibit prostate cancer cell proliferation, reduce androgen activity, and suppress pro-inflammatory cytokines, potentially lowering the prostate cancer risk [31,32].

Also, the notable cytotoxic effect on colorectal cancer HT29 cells should be highlighted, suggesting that further studies should be performed.

### 3.5. Anti-Inflammatory Activity of WGO

**Figure 3:**
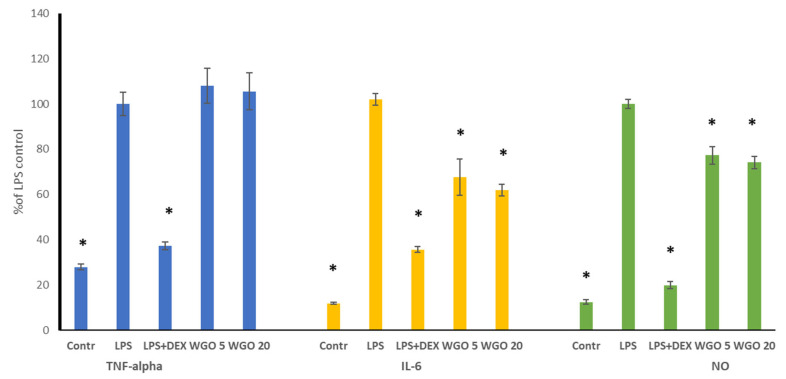
Effect of wheat germ oil (WGO) on TNF-alpha, IL-6, and NO release in LPS-stimulated RAW 264.7 macrophages. RAW cells were pre-treated with 5 (WGO 5) and 20 (WGO 20) µg/mL of oil for 1 h and then incubated with (10 ng/mL) or without LPS (untreated) overnight, in relation to dexamethasone (DEX) as a reference. Values are presented as the mean ± SD (standard deviation) of three independent experiments. The significant differences (*p* < 0.05) in each parameter in comparison to LPS are marked with asterisks (*).

Inflammation often accompanies various diseases, as it enables the promotion of cancer progression. Thus, encouraged by the results of the cytotoxic potential of WGO, we decided to complete our study with the determination of its anti-inflammatory properties, using a murine macrophage RAW 264.7 model stimulated by LPS to induce inflammation. To maintain appropriate assay performance, the effect of the WGO on the macrophage viability was tested first, and only two subcytotoxic doses were chosen for the assay, namely, 5 and 20 µg/mL. The results of the anti-inflammatory effects of WGO are displayed in Figure 3. For TNF-alpha, none of the doses of WGO reduced its release compared to the LPS control. In contrast, WGO significantly decreased the IL-6 production relative to the LPS control. Interestingly, the inhibition levels of IL-6 were similar for both doses of WGO, suggesting that increasing the dose from 5 to 20 µg/mL did not markedly enhance its effect on IL-6 production. For NO, WGO significantly reduced its production compared to the LPS control, with comparable levels of inhibition observed between the two doses.

These findings demonstrated that wheat germ oil does not have a dose-dependent anti-inflammatory effect, as evidenced by the significant reductions in IL-6 and NO production. A higher dose (20 µg/mL) showed a no stronger response, suggesting that the anti-inflammatory effects of WGO may plateau at higher concentrations. These results highlight the potential of WGO as a natural anti-inflammatory agent and suggest that further optimization of its dosing may help maximize its therapeutic benefits.

Only a few studies so far have reported the anti-inflammatory activity of WGO. Choi et al. [33] demonstrated that WGO suppressed the release of LPS-induced nitric oxide (NO) and pro-inflammatory cytokines, including IL-6, TNF-α, and IL-1β. Notably, an over 90% inhibition of IL-6 and TNF-α release was observed at a concentration of 100 μg/mL of the oil. WGO also inhibited the LPS-induced expression of cyclooxygenase-2, inducible nitric oxide synthase (iNOS), and nuclear factor-kappa B (NF-κB). Furthermore, it decreased the expression of phosphorylated ERK and JNK, which suggests that the anti-inflammatory effect of WGO was probably due to the modulation of NF-κB and the JNK/ERK MAPK signaling pathway. Zargar et al. [9] indicated, using in silico tools, that fatty acids from WGO exhibit significant anti-inflammatory activity through FABP4 binding and the regulation of PPARα, PPARγ, LPL, LEP, and ADIPOQ gene expression.

### 3.6. Human Serum Albumin Binding Potential

**Figure 4:**
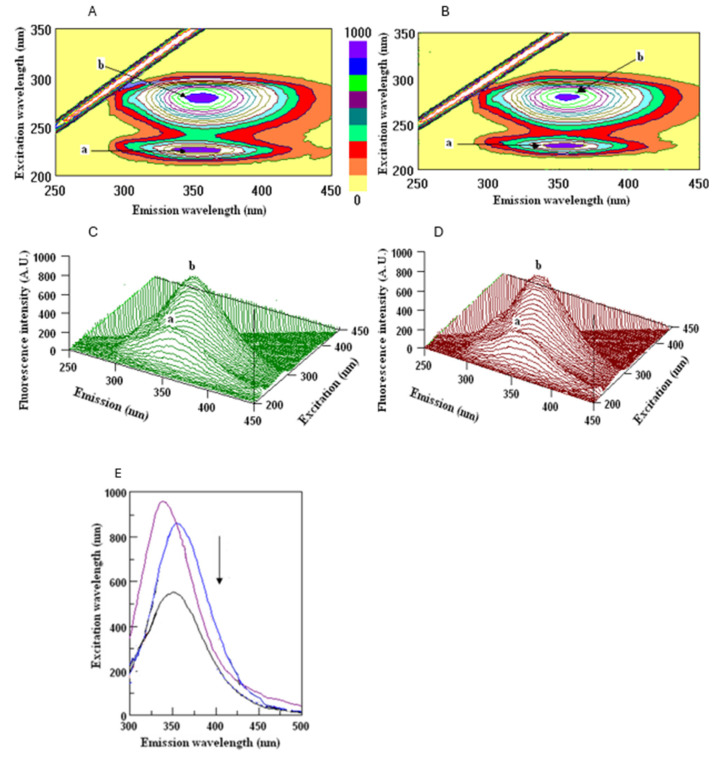
Counter maps (**A**,**B**) and their three-dimensional fluorescence (3D-FL) spectra (**C**,**D**) of interaction of human serum albumin (HSA) with Tween drop + NaCl (**A**,**C**) and with polyphenol extracts of wheat germ oil (WGO) (**B**,**D**); two-dimensional (2D-FL) fluorescence intensities (FIs) from the top (**E**): 1, HSA in buffer (purple line); 2, HSA + NaCl (blue line); 3, HSA + WGO extract (black line) with λem (nm) of 355, 354, and 354; FIs of 960.01, 862.18, and 787.01 arbitrary units. The polyphenols were extracted from 250 µL of oil with a mixture of 35 µL of Tween 80 and 4.5 mL of NaCl.

Even though the FTIR spectral analysis confirmed that WGO is predominantly a source of lipids, particularly triglycerides and squalene, with minimal contributions from polyphenols or flavonoids, the evaluation of the interaction of the polyphenolic fraction with HSA showed interesting results (Figure 4).

The interaction of human serum albumin (HSA) with Tween and NaCl solution, as well as with the polyphenol fraction of wheat germ oil (WGO), revealed significant differences in the fluorescence intensities and binding properties. The three-dimensional fluorescence (3D-FL) spectra demonstrated a notable decrease in the fluorescence intensity (FI) when HSA interacted with the polyphenol fraction, compared to its interaction with Tween and NaCl solution (Table 3). This indicates the formation of a stable HSA–polyphenol complex, suggesting a strong binding affinity. The extraction of polyphenols from WGO, using a Tween 80 and NaCl mixture, resulted in a binding peak of 19% (peak a = 16.2%, peak b = 2.8%).

These results underscore the high bioactivity of WGO polyphenols compared to that of other oil samples. This high binding percentage may be attributed to the antioxidant and protein-binding properties of polyphenolic compounds, which enhance the interaction with serum albumin [34]. Additionally, the observed FI reduction aligns with previous findings, where polyphenol–protein interactions quenched fluorescence, indicating structural changes or complex formation [35,36].

These findings highlight the potential of wheat germ oil as a bioactive oil with health-promoting properties. The high binding properties of its polyphenol content could make it a valuable component in functional foods and nutraceuticals. Further studies are recommended to explore its bioavailability and mechanisms of interaction with biomolecules.

## 4. Conclusions

The results demonstrate that WGO is a rich source of essential fatty acids, particularly linoleic acid (omega-6), with substantial contributions from oleic acid. The presence of α-linolenic acid (omega-3) further enhances its nutritional value. The squalene content and robust antioxidant activity highlight the oil’s potential as a functional ingredient in food, cosmetics, and nutraceuticals. Additionally, the substantial polyphenol content supports its role in mitigating oxidative stress and promoting health.

WGO demonstrated interesting, promising chemopreventive potential, directed particularly at skin and gastrointestinal cancer, supported by its anti-inflammatory properties and high safety. However, the potential role of WGO in supporting prostate cancer prevention should also be highlighted.

In summary, wheat germ oil represents a valuable, multi-faceted bioactive resource with diverse applications across health, wellness, and chemoprevention fields. Further exploration of its bioactive components and mechanisms of action will unlock new possibilities.

## Figures and Tables

**Table 1:** Fatty acid profile, squalene and total polyphenols, and antioxidant activity estimated as CUPRAC in wheat germ oil (WGO), (n = 3).

	**Fatty Acids Profile [%]**
Palmitic acid C16:0	19.4 ± 2.1
Stearic acid C18:0	4.6 ± 0.7
Oleic acid C18:1 n-9	26.8 ± 3.7
Linoleic acid C18:2 n-6	45.3 ± 5.1
α-linolenic acid C18:3 n-3	3.9 ± 0.6
	**Squalene [g/100 g]**
	2.52 ± 0.32
**Antioxidant activity**	**CUPRAC [** **μ** **M TE/mL]**
WGO	0.38 ± 0.07
Lipophilic fraction of WGO	0.13 ± 0.05 *
Hydrophilic fraction of WGO	0.24 ± 0.04 *
**Total polyphenols**	**TPC [µg GAE/mL]**
WGO	7.40 ± 0.80
Lipophilic fraction of WGO	2.70 ± 0.40 *
Hydrophilic fraction of WGO	4.69 ± 0.70 *

The significant differences (*p* < 0.05) between the hydrophilic and lipophilic phases are marked with an asterisk (*).

**Table 2:** Cytotoxic activity of wheat germ oil (WGO) and doxorubicin (reference drug) to normal and cancerous skin, prostate, and gastrointestinal cells after 24 h incubation, expressed as IC_50_ values (µg/mL).

Type of Cells	WGO	Doxorubicin
	Skin panel	
HaCaT	>Cmax	3.20
HTB140	37.2	4.45
A375	18.0	0.37
	Prostate panel	
PNT2	>Cmax	0.99
Du145	82.5	2.29
PC3	52.3	>40
LNCaP	15.4	3.48
	Gastrointestinal panel	
HT29	25.2	1.04
HepG2	62.4	1.11
Caco-2	>Cmax	2.79

Cmax was the highest concentration tested in the experiment.

**Table 3:** Fluorescence results of 3D measurements of wheat germ oil (WGO) in interaction with human serum albumin (HSA).

Sample	Peak a		Peak b	
	λ_ex_/λ_em (nm/nm)_ Int F_0_		λ_ex_/λ_em (nm/nm)_ Int F_0_	
HSA + water	228/347	746.30	280/350	854.34
HSA + Tween + NaCl	227/347	444.43	280/355	822.00
HSA + WGO	227/355	372.34	280/358	799.00

Abbreviations: Int F_0_—fluorescence intensity.

## Data Availability

The raw data supporting the conclusions of this article will be made available by the authors on request.
